# A novel single-lead percutaneous approach for multi-nerve peripheral stimulation in upper extremity pain: A case report

**DOI:** 10.1016/j.inpm.2025.100546

**Published:** 2025-01-31

**Authors:** Ryan S. D'Souza, Nasir Hussain

**Affiliations:** a Department of Anesthesiology and Perioperative Medicine, Mayo Clinic, Rochester, MN, USA; b Department of Anesthesiology, The Ohio State Wexner Medical Center, Columbus, OH, USA

**Keywords:** Peripheral nerve stimulation, Neuromodulation, Brachial plexus, Chronic pain

## Abstract

Peripheral nerve stimulation (PNS) is an emerging modality for managing painful peripheral neuropathy, offering potential long-term relief when conservative treatments fall short. Conventionally, each PNS lead targets a single nerve, necessitating multiple leads in cases involving pain across multiple nerve distributions. This case report presents a novel approach using a single PNS lead to target multiple peripheral nerves in the upper extremity via an axillary brachial plexus approach. We describe a 47-year-old female with a three-year history of intractable neuropathic pain localized to the ulnar and median nerve distributions, who underwent a temporary PNS trial after failing conventional therapies, including physical therapy, medications, and corticosteroid injections. Under ultrasound guidance, a single PNS lead was placed at the brachial plexus, targeting both ulnar and median nerves. Optimal stimulation thresholds were achieved, and the patient reported 80–100% pain relief throughout the 60-day trial period, with sustained relief for six months post-lead removal. This approach leverages the anatomical proximity of the ulnar, median, and radial nerves at the axilla, enabling multi-nerve targeting with a single lead. The technique offers potential advantages, including reduced procedural complexity, fewer risks, and cost savings, especially in the current landscape of increasing insurance denials for neuromodulation procedures.

## 1 Introduction

Painful peripheral neuropathy is a challenging condition to treat, especially when conservative measures including physical therapy, analgesics, and interventional treatments such as corticosteroid injections fail. While corticosteroid injections may provide localized relief, they are associated with side effects and diminishing efficacy over time [1]. Peripheral nerve stimulation (PNS) has emerged as an effective treatment modality, with data supporting intermediate and long-term relief with minimal adverse effects [[2], [3], [4]].

PNS delivers electrical impulses to peripheral nerves, alleviating pain via several hypothesized mechanisms, including the gate control theory, wide-dynamic range neuron modulation, and central modulation [5]. Durable relief has been demonstrated in both temporary and permanent PNS, with efficacy documented for up to 24 months [[6], [7], [8]]. Further, evidence suggests that implantable PNS devices may provide meaningful relief in peripheral neuropathic pain syndromes [9,10]. Unlike spinal cord stimulation (SCS), which spans multiple spinal segments, PNS requires precise lead placement near the peripheral nerve to stimulate a local area.

In conventional practice, each PNS lead targets a single nerve, limiting its utility in patients whose pain spans multiple nerve distributions. Such cases require the placement of multiple leads, increasing procedural complexity, risk, and cost. However, this case report describes a novel approach that utilizes a single PNS lead to target multiple peripheral nerves in the upper extremity via an axillary brachial plexus approach in a patient with refractory upper extremity neuropathic pain. To our knowledge, this is the first report describing this approach.

Overall, the objective was to demonstrate the feasibility of using a single PNS lead to stimulate multiple nerves in the upper extremity, and to describe the durability of effective pain relief achieved in a patient with painful peripheral neuropathy spanning multiple nerve distributions who had exhausted conventional treatment options. The patient's consent was obtained to disclose case details.

## 2 Case report

A 47-year-old female presented with a 3-year history of intractable neuropathic pain initially localized in the ulnar nerve distribution of her right upper extremity. Eleven years prior, she had undergone a cervical diskectomy at the C6-7 level for right-sided C7 radiculopathy, which treated her radicular pain for several years. On presentation to the pain clinic, she described her pain as stinging, sharp, and lancinating, with associated dysesthesias along the medial aspect of the right arm, forearm, and elbow, extending to the 4th and 5th digits of her hand. Her pain intensity ranged from 6 to 8 out of 10 on most days, occasionally escalating to 10/10 with activity. Her pain was exacerbated by activities involving elbow flexion and relieved by extending or shaking her arm. Exacerbation at night time and activities like driving or vacuuming were particularly bothersome, and she often wore a wrist splint for relief. She denied any weakness or numbness aside from her right upper limb symptoms, neck pain, or difficulties with balance, or bowel or bladder incontinence. Physical examination revealed hyperalgesia and diminished sensation to touch along the medial aspect of her right forearm and elbow. There were no sudomotor, vasomotor, or trophic changes. She had 5/5 strength bilaterally with wrist flexion and extension, elbow flexion and extension, and forearm supination and pronation.

Initially, there was concern for recurrent C7 radiculitis or a right ulnar mononeuropathy, though EMG studies revealed evidence of mild right carpal tunnel syndrome and a chronic right C7 radiculopathy without active denervation. Imaging revealed a moderate right-sided disc protrusion at C6. The patient had trialed physical therapy as well as multiple analgesics including ibuprofen 400 mg as needed, gabapentin 300 mg three times daily, and duloxetine 60 mg nightly, which did not provide any meaningful relief. The patient also underwent a series of treatments, including a right paramedian interlaminar C7-T1 epidural steroid injection which provided no relief. However, a right ulnar nerve corticosteroid injection at the elbow provided substantial temporary relief lasting 3–4 months. Over the course of three years, she had received 11 ulnar nerve corticosteroid injections, which offered 80–100 % pain relief but ultimately resulted in significant skin and subcutaneous tissue atrophy near the cubital tunnel. The atrophy was notable and given how superficial the ulnar nerve is at the cubital tunnel area, the lack of subcutaneous tissue at this region was concerning to the patient who then reported constant dysesthesias with even the slightest touch or pressure along her medial elbow.

Given the substantial tissue atrophy and diminishing efficacy of corticosteroid injections, the patient elected to pursue a temporary PNS trial. At the time of PNS implantation, physical examination revealed that her pain had expanded to include the median nerve distribution in addition to the ulnar nerve. This new pain in the median nerve distribution may also suggest worsening carpal tunnel syndrome. Therefore, the decision was made to place a single PNS lead proximally at the brachial plexus via the axillary approach to target both the ulnar and median nerves. This was chosen to avoid placing separate leads near the elbow, where subcutaneous tissue was limited, and the patient's pain extended proximally beyond the elbow.

**Fig. 1:**
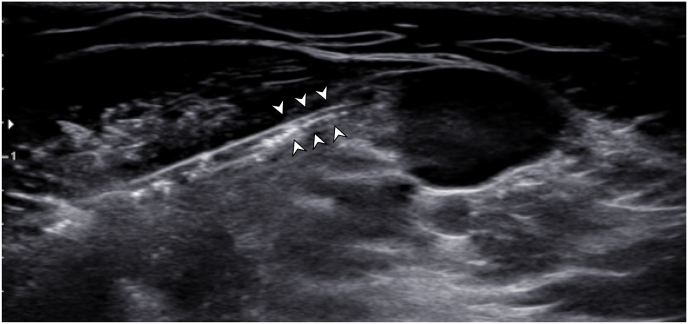
Ultrasound image displays the introducer needle approaching the region between the median nerve and ulnar nerve.

Under ultrasound guidance via the axillary brachial plexus approach, the axillary artery was visualized, along with the various peripheral nerves that circumferentially lie around the artery (ulnar, median, and radial nerves). A 19-gauge stimulating probe was advanced a certain distance beyond the 17-gauge percutaneous sleeve utilizing an in-plane technique to a target position adjacent to both the ulnar and median nerve (Fig. 1). Of note, care was taken to assure that local anesthetic was not administered close to the nerve targets to prevent an altered response to test stimulation. Once the needle was positioned between the median and ulnar nerve targets, test stimulation was performed by connecting a manufacturer-provided test cable (SPR Therapeutics, Cleveland, OH, USA) to the probe. Intensity was adjusted based on patient feedback until comfortable paresthesias from the stimulator encompassed the regions of pain, including both the median and ulnar nerve distributions.

Once an optimal location and stimulation threshold were identified, the test cable was disconnected from the stimulating probe and the probe was subsequently removed from the sleeve. The sleeve was held in place while a 20-gauge Microlead Introducer, containing the PNS lead to be implanted, was inserted and advanced to the target depth. The connector box was then attached to the distal de-insulated lead and its associated cable was connected to the stimulator. Test stimulation was again conducted with adjustments made until the desired response was obtained based on patient feedback. Finally, the introducer-sleeve apparatus was carefully withdrawn from the skin while manual traction and pressure were applied at the lead entry site. Removal of the introducer-sleeve apparatus deploys the anchor, which consists of an acute bend at the distal 5 mm of the lead tip; this curve in the lead provides purchase in the tissue while remaining flexible to enable lead removal after treatment.

Ultrasound-guided confirmation was obtained to identify that the single temporary PNS lead was positioned between the median and ulnar nerves, and test stimulation was performed to confirm comfortable sensory paresthesias in both nerve distributions. Finally, the external pulse generator (EPG) was connected to the PNS lead, and the EPG was positioned away from the lead-entry site in a comfortable position for the patient. The manufacturer of the PNS device was SPR Therapeutics (Cleveland, OH, USA). The lead has a single active electrode contact, and sensory stimulation was delivered at 100 Hz. The patient was able to adjust the intensity within the full range of amplitude (0.2–30 mA) and pulse width (10–200 μs). The patient was counseled to use the PNS device for at least 12 hours daily, but the device can be used for up to 24 hours daily.

Post-procedurally at day 2, the patient reported significant improvement in her neuropathic pain, with 80–100% pain relief across both the ulnar and median nerve distributions. She experienced comfortable paresthesias with stimulation, and her pain was well-controlled throughout the 60-day trial period. At the end of the trial, she reported over 80% symptom relief, which was sustained for six months post-removal of the temporary PNS lead. Concordantly, she also reported 70–80% improvement in physical function, highlighting her ability to resume activities such as vacuuming and carrying her granddaughter—tasks she had previously been unable to perform. Her physical examination was unchanged compared to prior PNS placement. The patient is currently being evaluated for permanent PNS implantation using this aforementioned novel approach.

## 3 Discussion

This case highlights a novel approach of using a single PNS lead to target multiple peripheral nerves in the upper extremity. Conventionally, each PNS lead targets a single nerve, requiring multiple leads in cases where pain spans more than one peripheral nerve distribution. By placing the lead via an axillary brachial plexus approach, stimulation of both the ulnar and median nerves with a single lead was feasible, offering significant pain relief without the need for multiple procedures or hardware.

**Fig. 2:**
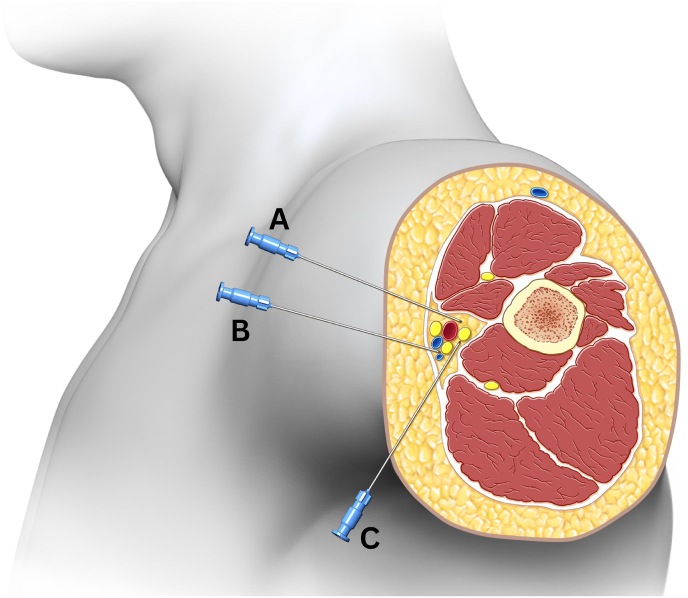
Diagram demonstrates various needle trajectories for peripheral nerve stimulator lead placement. Needle trajectory “A” would result in perpendicular lead placement adjacent to the median and radial nerve. Needle trajectory “B” would result in perpendicular lead placement adjacent to the median and ulnar nerve. Needle trajectory “C” would result in perpendicular lead placement adjacent to the ulnar and radial nerve.

Anatomically, the close proximity of the ulnar, median, and radial nerves at the level of the axilla makes this technique feasible. Depending on lead placement, it may be possible to target various nerve combinations, including the ulnar and median nerves (as in this case), the radial and median nerves, or the ulnar and radial nerves. The potential needle trajectories targeting each of these nerve combinations are displayed in Fig. 2. Additionally, the musculocutaneous nerve, which lies within the coracobrachialis muscle, could potentially be targeted in future cases involving more diffuse pain distributions.

This technique offers several advantages, including reduced procedural complexity and potential cost savings. By targeting two nerves with a single lead, this approach minimizes the number of needle insertions and hardware required, which is particularly beneficial in the context of increasing insurance denials and decreased reimbursement for PNS and neuromodulation procedures. Patients with more diffuse neuropathic pain syndromes, such as those with complex regional pain syndrome (CRPS), painful diabetic neuropathy, or chemotherapy-induced peripheral neuropathy (CIPN), could benefit from a multi-nerve targeting approach with potential for fewer procedural risks from less needle insertions. Further studies are warranted to explore the long-term efficacy and safety of this approach, as well as its applicability in other anatomical regions and pain syndromes.

In conclusion, we report a novel approach for PNS in the upper extremity using a single PNS lead to target both the ulnar and median nerves. This technique demonstrated significant and durable relief in a patient with refractory neuropathic pain, with no complications. Future studies are warranted to further validate this technique and explore its broader clinical applications.

## 4 Author contributions

Ryan D'Souza (study conception, manuscript composition, data validation, data extraction and analysis, supervision of all aspects of the study), Nassir Hussain (data analysis and validation, editing and supervision of study)

## Funding

The authors have not declared a specific grant for this research from any funding agency in the public, commercial or not-for-profit sectors.

## Declaration of competing interest

RSD received investigator-initiated research grant funding from Nevro Corp and Saol Therapeutics paid to his institution. Other authors declare no conflict of interest.
